# Novel Boron–Arginine Derivatives Induce Apoptosis and Exhibit Anti-Inflammatory and Antimicrobial Activities in B16-F10 Melanoma Cells

**DOI:** 10.1007/s12011-026-05172-9

**Published:** 2026-06-06

**Authors:** Meliha Koldemir-Gündüz, Aynabat Esenjanaova, Elif Aydın, Güllü Kaymak, Ayşe Koçak-Sezgin, Dursun Ali Köse

**Affiliations:** 1 Department of Basic Sciences of Engineering, Faculty of Engineering and Natural Sciences, Kutahya Health Sciences University, Kutahya, Türkiye; 2 Department of Chemistry, Faculty of Arts and Sciences, Hitit University, Çorum, Turkey; 3 Department of Medical Microbiology, Medical Faculty, Agri Ibrahim Cecen University, Agri, Türkiye; 4 Simav Vocational School of Health Services, Kütahya Health Sciences University, Kütahya, Turkey; 5 Department of Medical Biochemistry, Faculty of Medicine, Kutahya Health Sciences University, Kutahya, Türkiye

**Keywords:** Boron arginine monoester, Boron arginine diester, New synthesis, Melanoma, Apoptosis, Anti-inflammatory

## Abstract

Melanoma is an aggressive form of cancer characterized by rapid progression and resistance to conventional therapies, highlighting the need for novel therapeutic agents with low toxicity and multifunctional activity. This study evaluated the potential effects of newly synthesized boron arginine monoester (BAM) and boron arginine diester (BAD) in B16-F10 melanoma cell line. Following 48 h of incubation, the IC₅₀ values of BAM and BAD were 5.97 mM and 51.9 µM, respectively, suggesting relatively low cytotoxic effects under the tested conditions. Both compounds were associated with changes in apoptosis-related markers, such as an increased Bax/Bcl-2 ratio and upregulation of p53 expression, suggesting a possible involvement of apoptosis-related processes. In addition, they exhibited modulatory effects on cytokine levels, including TNF-α, IL-6, and IL-10, depending on the treatment conditions. BAM and BAD exhibited antimicrobial activity against both Gram-positive and Gram-negative bacteria (MIC: 3.12–12.5 µg/mL; MBC: 12.5–50 µg/mL). These findings are presented for completeness, while the primary focus of this study remains on the compounds’ effects on melanoma cells, apoptosis, and cytokine modulation. Overall, these findings indicate that BAM and BAD possess apoptotic, anti-inflammatory, and antimicrobial properties, suggesting their potential as multifunctional agents in melanoma treatment. Further studies are required to elucidate their mechanisms of action in different models and to evaluate their long-term therapeutic efficacy.

## Introduction

Malignant melanoma (MM) is a highly aggressive tumor arising from the malignant transformation of melanocytes, primarily affecting the skin and mucosal tissues. Although most cases occur in the skin, MM may also develop in regions where melanocytes migrate during embryogenesis, including the eyes, oral and genital mucosa, and leptomeninges [1, 2]. Despite substantial improvements in cancer screening, early detection, and therapeutic strategies that have reduced the incidence of many cancers, both the incidence and mortality of MM have risen markedly over the last 20 years, especially in industrialized countries [1, 3]. This rise cannot be attributed solely to improved detection, but also reflects a true increase in disease incidence [4]. According to GLOBOCAN 2020 data, approximately 325,000 new melanoma cases and 57,000 melanoma-related deaths were reported globally, with the highest incidence observed in Australia (> 37 per 100,000 individuals annually) [5]. Ultraviolet (UV) radiation remains the primary etiological factor, while additional risk factors include fair skin, multiple pigmented nevi, age, gender, genetic predisposition, and environmental influences [6, 7]. Reactive oxygen species (ROS) are also known to contribute to melanoma progression by promoting melanocyte proliferation and metastasis [8].

Current treatment strategies for MM include chemotherapy, radiotherapy, immunotherapy, and targeted therapies tailored to specific genetic mutations. However, these approaches are often limited by drug resistance, low tissue selectivity, and significant toxicity [9, 10]. Moreover, the prognosis remains poor in metastatic cases, with a 5-year survival rate of approximately 5–10% following surgical intervention [11]. These limitations highlight the urgent need for the development of novel and more effective therapeutic strategies.

Boron, a biologically relevant trace element, is involved in multiple physiological functions and is indispensable for normal growth and development across plant, animal, and human systems [12–14]. It is rapidly absorbed and distributed throughout the body, influencing various metabolic processes [15]. Boron-containing compounds, such as boric acid and borax, have demonstrated diverse biological activities, including anticarcinogenic, anti-inflammatory, antimicrobial, and antioxidant effects [16–18]. However, the molecular mechanisms of the cytotoxic effects of boron compounds have not yet been fully elucidated, and recent studies have attempted to address this gap in the existing literature [14, 18–22]. Thus, understanding the cellular alterations brought about by recently synthesized boron compounds is crucial for future anticancer research, as it will guide in identifying their potential therapeutic benefits and further investigations in cancer research.

It is known that boron taken in the form of boric acid is hydrolyzed to tetrahydroxyborate anion in the small intestine after being taken into the body, and the majority a fraction of the boron-based compound is excreted unchanged in the urine. A certain amount of it accumulates in body tissues such as spleen and bone, which is an undesirable situation [21]. In recent years, boron ester–based compounds have gained considerable attention as dietary supplements, which possess increased hydrolytic stability and enhanced cellular internalization due to the presence of their bioactive ligands It is a well-known fact that boron influences bone metabolism, particularly by reducing the urinary excretion of Ca^2^⁺ and Mg^2^⁺ ions. Boron has important biological roles, and recent studies highlight its potential relevance in cancer, particularly melanoma. These properties may provide advantages in terms of bioavailability and therapeutic potential. Despite growing interest in boron-based compounds, their precise roles in cancer biology and metabolism remain to be fully elucidated [19, 20, 22–24]. Boron-containing compounds have gained significant attention in cancer research due to their potential cytotoxic effects and selective targeting properties. In this study, arginine was incorporated into the boron ester derivatives to enhance their biocompatibility and cellular uptake. The positively charged guanidino group of arginine facilitates interaction with the negatively charged cell membrane, potentially increasing the internalization of boron compounds and thereby improving their cytotoxic efficacy toward melanoma cells. These boron-arginine mono- and diesters (BAM and BAD) were designed to evaluate their antiproliferative effects on B16-F10 melanoma cells.

In this present study, BAM and BAD bioactive molecules were synthesized and investigated for their therapeutic potential against MM. Further, the antibacterial, antioxidant, and anticancer activities of the synthesized boron-based compounds were evaluated using B16-F10 melanoma, a cellular model.

## Materials and Methods

This study was conducted entirely in vitro, and no experimental animals were used. Biological evaluations were performed on the murine B16-F10 melanoma cell line, commonly used in melanoma research. This cell line is frequently preferred in experimental melanoma models due to its rapid proliferation capacity and invasive properties. However, it was considered that this cellular model does not fully represent human melanoma, and the findings obtained cannot be directly generalized to clinical outcomes. This limitation was taken into account in the interpretation of the study.

### Synthesis of Boron Arginine Monoester and Boron Arginine Diester

Boron-arginine mono- and diesters (BAM and BAD) were synthesized by incorporating arginine into the boron moiety, leveraging arginine’s cell-penetrating properties and biocompatibility. This design aimed to facilitate efficient delivery of the boron compounds into B16-F10 melanoma cells, enhancing their cytotoxic activity.

Arginine (Sigma-Aldrich) was dissolved in distilled water and methanol (Sigma-Aldrich). Solid H_3_BO_3_ (Sigma-Aldrich) was then added, and the reaction mixture was stirred magnetically at room temperature for approximately 1 h. The solution was allowed to crystallize at room temperature after some of the solvents were removed from the evaporator until it thickened. In a vacuum oven, the finished product was dried at 50 °C. It was then characterized using elemental analysis, FT-IR spectroscopy, melting point determination, and thermal analysis methods [25].

It was then characterized using elemental analysis, FT-IR spectroscopy, melting point determination, and thermal analysis methods. Specific molar ratios, pH, yields, and conditions distinguishing mono- versus diester formation were optimized experimentally. Full structural confirmation, including ^11^B NMR spectroscopy, is recommended for comprehensive characterization of the boron esters.

### Cell Culture

The mouse B16-F10 cells utilized in the investigation were obtained from the American Type Culture Collection (ATCC) in Manassas, USA. B16-F10 melanoma cells (1 × 10⁶ cells were seeded per 25 cm^2^ flask) were cultured in high-glucose DMEM (4.5 g/L glucose) supplemented with 10% FBS and 1% penicillin–streptomycin, under standard conditions (37 °C, 5% CO₂). High-glucose medium was used to support optimal cell proliferation. BAM and BAD treatments were applied to these cultures, and the glucose content of the medium is not expected to interfere with their cytotoxic activity. Cells were seeded at a density of 1 × 10⁶ per 25 cm^2^ flask and allowed to adhere for 24 h before treatment with the compounds. Every two to three days, the medium was replaced in a humidified setting at 37^0^C with 5% CO_2_. Sub-cultured as needed using a standard technique of trypsinization [16].

### Cytotoxicity Testing with RTCA iCELLigence™

For the B16-F10 melanoma cell growth studies, the ideal cell concentration was established. After the cells were seeded in 150 μL of medium into each well of E-plate 8, the proliferation of the cells was monitored by the iCELLigence system every 15 min. Boron-arginine mono ester and boron-arginine diester solutions were freshly prepared in the medium. After the cells adhered to the plate bottom and reached the required density, they were exposed to 150 μL of medium containing 50 mM, 10 mM, 5 mM, 2.5 mM, 1 mM, 500 μM, 250 μM BAM and BAD substances. The control cell group received only culture medium [16].

IC₅₀ values were calculated using the iCELLigence™ system software based on the sigmoidal dose–response curve model at specific time points (24 h and 48 h). The IC₅₀ represents the concentration of compound required to inhibit 50% of cell proliferation relative to the untreated control.

### RNA Isolation and qPCR Examination

Total RNA was isolated from B16-F10 cells using RNeasy Mini Kit (Qiagen standard) according to the manufacturer’s instructions. Samples of total RNA were stored at −80 °C for further use. RNA purity was assessed by measuring A260/A280 ratios, and RNA integrity was verified. For cDNA synthesis, 1 µg of total RNA was reverse-transcribed in a 20 µL reaction using Transcriptor HiFi cDNA Synthesis Kit, Roche. qPCR to evaluate the expression of the genes for TNF-α, IL-6, Bax, Bcl-2, and p53. qPCR was performed using ABI StepOnePlus (Applied Biosystems, Germany) cycling parameters of initial denaturation at 95 °C, 5 min, followed by 40 cycles of 95 °C for 10 s, 60 °C for 30 s, 72 °C for 30 s. Primer efficiencies were determined and were within the acceptable range of 90–110%. All experiments included no-RT controls, no-template controls, and positive controls to ensure specificity and accuracy of the qPCR results. For every sample under analysis, at least three replicates were carried out. The mathematical computations for Ct ≥ 40 excluded. Expression levels were calculated in relation to the reference gene's (GAPDH) expression. To verify the existence of particular gene products, the 2^−ΔΔCt^ method was employed. Primers were: TNF-α (forward) 5′-TCG TAG CAA ACC ACC AAG TG-3′, (reverse) 5′-CGG ACT CCG CAA AGT CTA AG-3′; IL-6 (forward) 5′-GCA AGA GAC TTC CAT CCA GTT G-3′, (reverse) 5′-ACT CCA GGT AGC TAT GGT ACT CCA-3′; Bax (forward) 5′-TGC AGA GGA TGA TTG CCG CCG-3′, (reverse) 5′-ACC CAA CCA CCC TGG TGT TGG-3′; Bcl-2 (forward) 5′-CCT GTG CAC CAA GGT GCC GGA ACT-3′, (reverse) 5′-CCA CCC TGG TCT TGG ATC CAG CC-3′; p53 (forward) 5′-GCC CAA CAA CAC CAG CTC CT-3′, (reverse) 5′- CCT GGG CAT CCT TGA GTT CC-3′; GAPDH (forward) 5′-AGG GCA AGA TGG CTG TAA GGT-3′ and (reverse) 5′-GGG ACC AAC TTC GGA ATC AG-3′.

### Biochemical Analysis

Following BAM and BAD treatments, B16-F10 cells were trypsinized from culture flasks and homogenized in an appropriate homogenization buffer (phosphate-buffered saline containing protease inhibitors, 1 mL per 10⁶ cells) using a mechanical homogenizer. (TissueLyser, Qiagen) All procedures were performed under cold conditions to maintain the cold chain. The homogenate was centrifuged at 10,000 rpm for 20 min at 4 °C, after which the pellet was discarded and the supernatant was collected for subsequent biochemical analyses. Each assay was performed in triplicate (n = 3) for each condition. Spectrophotometric measurements were carried out using a Thermo Scientific Evolution UV–Vis spectrophotometer.

Coomassie brilliant blue (G-250) dye was used to determine the total amount of proteins, and the study's methodology involved measuring the absorbance of the colored solutions that resulted from the dye's binding to the proteins at 595 nm [26].

Thiobarbituric acid (TBA) and the lipid peroxidation product MDA were utilized to determine malondialdehyde (532 nM). Spectrophotometric analysis was used to determine the absorbance of the pink/red chromogen that resulted from their reaction [27].

For total glutathione determination, Ellman's reagent was used. Spectrophotometric analysis was used to assess the colors that emerged from the reaction of DTNB (5–5'dithiobis 1–2 nitrobenzoic acid) with sulfhydryl groups. The reaction produced a yellow color, which was quantified by measuring the absorbance at 412 nm. [28]. Total antioxidant status (TAS) and total oxidant status (TOS) were measured using commercial enzyme-linked immunosorbent assay (ELISA) kits with well plates coated with immobilized antibodies specific to the target protein (Rel Assay Diagnostics TAS Kit, Turkey; Rel Assay Diagnostics TOS Kit, Turkey). The following formula was used to generate the Oxidative Stress Index (OSI) [29]. OSI (arbitrary unit) = TOS (μmol H2O2 Eq/L)/TAS (μmol Trolox Eq/L).

### Measurement of TNF-α, IL-1β, IL-6, and IL-10 Levels

ELISA was used to detect the levels of cytokine expression for: TNF-α (Reed Biotech, Wuhan, Hubei, China), IL-1β (Reed Biotech, Wuhan, China), IL-6 (Reed Biotech, Wuhan, Hubei, China) and IL-10 (Reed Biotech,Wuhan Hubei, China) (ELISA murine cytokine antibody kits—Reed Biotech, Wuhan, Hubei, China). The reaction produced a yellow color, which was quantified by measuring the absorbance at 412 nm using 100 µL of cell lysate, with three intra-assay replicates; measurements were performed on a Thermo Multiskan GO ELISA plate reader and normalized per mg protein [16].

### Determination of Annexin V and Bcl-2 Levels by ELISA

The levels of annexin V (SEA259Mu, Wuhan USCN Business Co., Ltd, China) and Bcl-2 (SEA778Mu, Wuhan USCN Business Co., Ltd, China) in cells were measured using 100 µL of cell lysate with three intra-assay replicates, following the kit protocol. Absorbance was read at 450 nm using a Thermo Multiskan GO ELISA plate reader. Results were normalized per mg protein. The assay’s intra-assay variability (CV) was determined; LOD and LOQ were as reported by the manufacturer [16].

### Antimicrobial Activity of BAM and BAD Compounds

The bacterial strains used in this study were selected from the American Type Culture Collection (ATCC) and are quality-controlled according to recommendations from the Clinical and Laboratory Standards Institute [30]. The following Gram-positive bacteria were included: Methicillin-resistant *Staphylococcus aureus* (MRSA) ATCC 43300, *Staphylococcus epidermidis* ATCC 35984, and *Enterococcus faecalis* ATCC 29212; Gram-negative bacteria included: *Acinetobacter baumannii* ATCC 19606, *Escherichia coli* ATCC 25922, *Proteus vulgaris* ATCC 6897, and *Klebsiella pneumoniae* ATCC 700603.

The minimal inhibitory concentration (MIC), defined as the lowest concentration of a compound that completely prevents bacterial growth in microdilution wells, was determined using standard microdilution assays. Dimethyl sulfoxide (DMSO) was used to dissolve BAM and BAD compounds (Merck & Co., Darmstadt, Germany) at a stock concentration of 10 mg/mL, and the final DMSO concentration in assay wells did not exceed 1% to avoid cytotoxic effects. Cation-adjusted Mueller–Hinton broth (CAMHB, HiMedia, India) was used as the growth medium.

Frozen bacterial stocks were first cultured on Tryptone Soya Agar (TSA) at 37 °C for 18–24 h. Single colonies were suspended in 0.9% NaCl and adjusted to 0.5 McFarland turbidity. For the assay, 50 µL of CAMHB was added to all wells, followed by serial two-fold dilutions of BAM or BAD compounds to generate concentrations ranging from 200 to 0.30 µg/mL. Fifty microliters of bacterial inoculum was added to each well.

Controls included:

Positive control: wells with inoculum only (growth expected).

Test wells: inoculum + compound.

Sterility control: medium only (no inoculum, no compound).

Plates were incubated at 37 °C for 24 h. Bacterial growth was assessed visually and confirmed by optical density at 600 nm (OD600). Each assay was performed in triplicate (n = 3). The MIC was recorded as the lowest concentration showing no visible growth. To determine the minimal bactericidal concentration (MBC), samples from wells showing no growth were sub-cultured on TSA plates; the MBC was defined as the lowest concentration showing no colony formation after incubation.

### Statistical Analysis

The iCELLigence system software was utilized to process experimental data, including calculation of logarithmic half-maximal inhibition concentration (log [IC50]) values by fitting a sigmoidal dose–response curve. Results are reported as mean ± standard deviation (SD). SPSS software (version 23.0; SPSS Inc., Chicago, IL, USA) was used for all statistical analyses. The following tests were applied: Two-group comparisons: Parametric comparisons were performed using Student’s t-test, and non-parametric comparisons were performed using the Mann–Whitney U test, depending on variance equality. Comparisons among more than two groups: One-way ANOVA was used, followed by Tukey’s post hoc test to identify pairwise differences. Correlation analyses: Spearman’s correlation test was used to assess associations between gene expression levels (e.g., *TNF-α, IL-6, Bax, Bcl-2, p53, GAPDH*). All experiments were conducted with the indicated biological and technical replicates (n = 3), as specified in each Methods subsection. Exact p-values are reported in the Results section (e.g., p = 0.012), and significance is indicated as p < 0.05, p < 0.01, etc.

## Results

### Synthesis

**Fig. 1:**
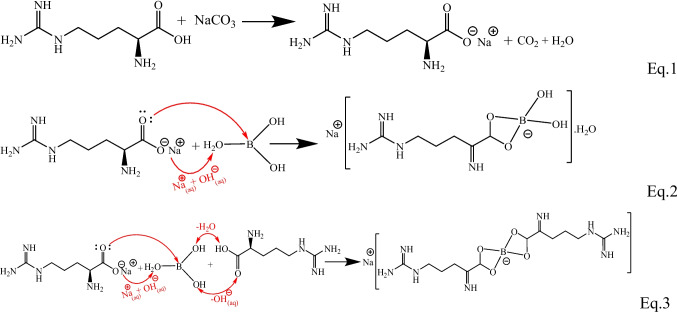
The synthesis equation

Boron is a weak Lewis acid containing multiple hydroxyl groups (cis-diols) and an empty p orbital, which enables the formation of tetrahedral anionic ester complexes with ligands possessing adjacent hydroxyl groups. In aqueous media, boric acid predominantly exists as the tetrahydroxyborate anion, B(OH)₄⁻, which can undergo esterification reactions with cis-diol-containing ligands. Depending on the stoichiometry, mono-chelate (1:1) and bis-chelate (1:2) complexes can be formed [21, 31, 32]. The formation of boron ester complexes is based on the interaction between the –OH groups of B(OH)₄⁻ and the hydroxyl groups of the ligand. Due to the negative charge on the boron center, metal cations are required to stabilize the resulting complexes; in this study, NaHCO₃ was used as the sodium source. Initially, arginine was converted into its sodium salt (Na-arginate) by reaction with NaHCO₃ (Eq. 1) (Fig. 1), with the release of CO₂ and H₂O. For the synthesis of boron arginine monoester (BAM), boric acid was added to the Na-arginate solution at a 1:1 molar ratio. During this process, coordination occurs via electron donation from the carbonyl oxygen to the empty p orbital of boron, accompanied by esterification between –OH groups of B(OH)₄⁻ and the ligand (Eq. 2) (Fig. 1). For the synthesis of boron arginine diester (BAD), a second equivalent of arginine was added, resulting in further esterification of the remaining –OH groups of boric acid and formation of a bis-chelate (1:2) structure (Eq. 3) [23, 24, 31] (Fig. 1). After completion of the reaction, the solvent was removed under reduced pressure using an evaporator. The resulting solution was treated with cold acetone to induce precipitation. The precipitated product was collected by vacuum filtration and immediately transferred to a desiccator to prevent moisture absorption. Ultrapure water used in the synthesis was pretreated by boiling for 2 h under a nitrogen atmosphere to remove dissolved gases. The chemical composition of the synthesized ester compounds is presented in Table 1.


1
2



3


**Table 1:** Results of boron-arginine ester compounds' elemental analysis

Complexes	M.W. (g/mol)	Yield	Content (%) Exp. (Teo.)			Colour	M.P (°C)
			**C**	**H**	**N**		
Na[B(OH)_2_(C_6_H_12_N_4_O_2_)].H_2_O C_6_H_16_BN_4_NaO_5_	258.02	73	27.28 (27.93)	6.73 (6.25)	21.61 (21.71)	White	126
Na[B(C_6_H_12_N_4_O_2_)_2_] C_12_H_24_BN_8_NaO_4_	378.18	69	38.91 (38.11)	6.80 (6.40)	29.51 (29.63)	White	122

To characterize the structural properties of the synthesized compounds, solid-state analyses were performed. Infrared (IR) spectroscopy was used to examine binding properties, while thermogravimetric (TGA) and differential thermal analysis (DTA) curves were recorded to evaluate thermal behavior. The data were compared with previously reported single-crystal structures of related boron ester compounds to infer molecular structure [25, 32–35].

**Fig. 2:**
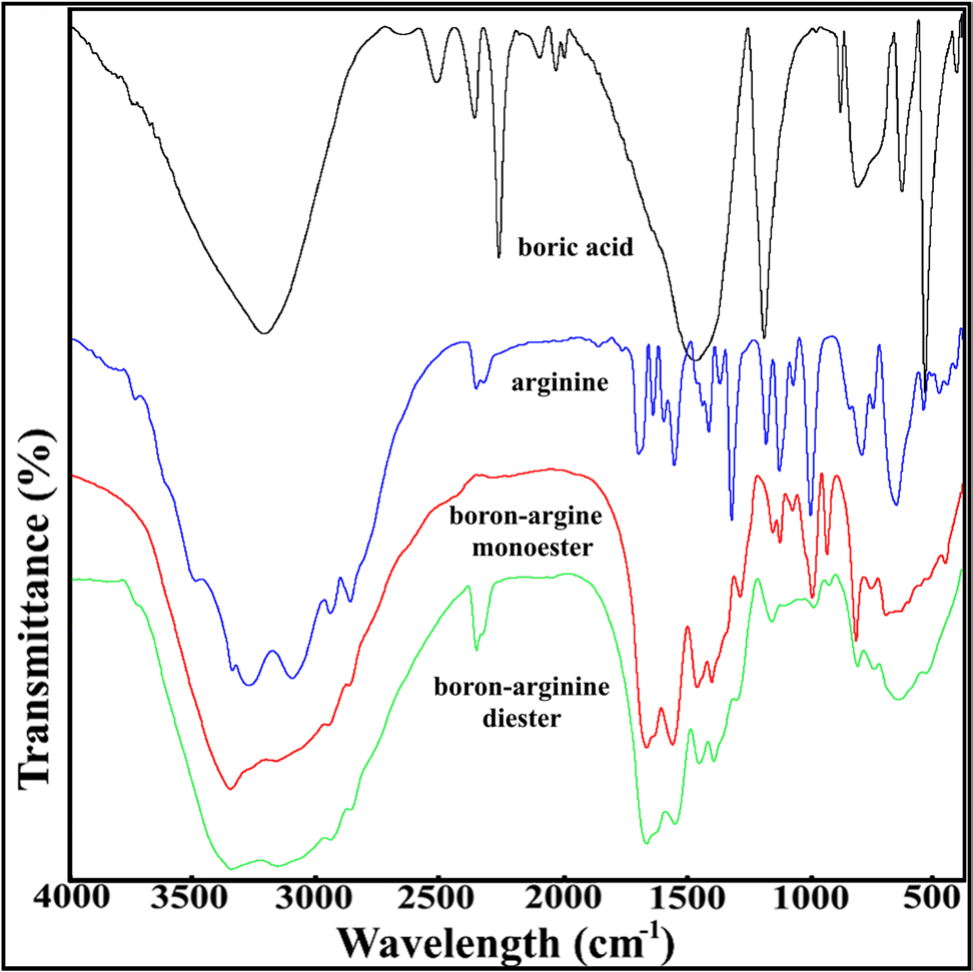
FT-IR spectra of boron-arginine ester compounds

In the IR spectra (Fig. 2), the ester bonds were identified through characteristic stretching vibrations: asymmetric νasym(BO)/BO₄ and ν(C–O), and symmetric νsym(BO)/BO₄. The asymmetric νasym(BO)/BO₄ stretching of boric acid at 1220 cm⁻^1^ shifted to 1162 cm⁻^1^ in BAM and 1167 cm⁻^1^ in BAD. Similarly, the symmetric νsym(BO)/BO₄ stretching at 834 + 815 cm⁻^1^ in boric acid shifted to 826 + 748 cm⁻^1^ in BAM and 826 + 756 cm⁻^1^ in BAD, consistent with ester bond formation.

The broad –OH peak observed in the diester compound is attributed to residual water in molecular cavities, likely due to incomplete drying. Additionally, the twin –NH stretching peaks of arginine at 2891 and 2808 cm⁻^1^ shifted to 2672 and 2678 cm⁻^1^ in BAM and BAD, respectively, indicating conversion of the acidic –NH₂ group to –NH following esterification. In the diester molecule, –NH stretching appeared as a shoulder beneath the broad –OH peak of the aqua ligand. All significant peak values are summarized in Table 2 and are consistent with the literature.

**Table 2:** Key infrared peaks of compounds containing boron-arginine esters

Molecules	ν(O–H)& ν(N–H)	ν(CN)& ν_a_(C = C)	ν_a_(COO)	ν_s_(COO)	ν_a_(BO)/BO_4_ & ν(C-O)&ν(N–H)	ν_s_(BO)/BO_4_
Arginine	3496**–**3293	1739**–**1686	-	-	1252	-
Boric acid	~ 3300		-	-	1220 & -	834 + 815
BAM	3354–2932	2672	1567	1408	1008**–**1162	826 + 748
BAD	3354–2982	2678	1563	1406	1002–1167	826 + 756

**Fig. 3:**
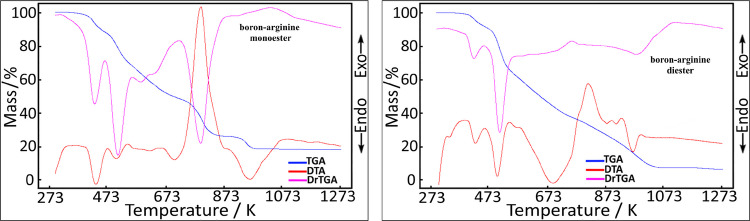
TGA/DTA/DrTGA curves of boron-arginine ester compounds

The thermal degradation of the molecules is summarized in Table 3, based on TGA, DTA, and DTGA curves (Fig. 3). While hydrate water is present in the BAM structure, it is absent in the BAD structure. The first step in BAM degradation involves the removal of hydrate water. Subsequent degradation steps proceed similarly for both compounds. The two hydroxy groups attached to boronate ester, boron center, or boron atom in the ester in the monoester are also released as water molecules. Following dehydration, the –NH group of the arginine ligand is eliminated, after which the remaining organic residue undergoes combustion and decomposition. For the diester compound, two degradation stages were observed, corresponding to the removal of –NH groups, followed by thermal decomposition of the organic residue. Powder XRD measurements confirmed that the residue remaining after thermal decomposition of both esters is NaBO₂. The experimental and theoretical outcomes for all decomposition steps are in good agreement. The slightly higher experimental residue compared to the theoretical value is attributed to the decomposition occurring under an inert nitrogen atmosphere. Limited oxygen availability prevents complete combustion, resulting in the formation of carbonized material on the residue, which changes its color from the predicted white to black [36–38].

**Table 3:** Data on the thermal breakdown of compounds containing boron-arginine esters

Ester molecules		Temp. Range (˚C)	DTA_max_ (˚C)	Removed Group	Mass Loss (%)		Remained Product (%)		Decomp. Product	Colour
					Exp	Calc	Exp	Calc		
Na[B(OH)_2_(arg)]⋅H_2_O	1	76–204	153	2H_2_O	13.12	13.95				white
BAM	2	205–290	228	3NH_3_	19.57	19.80				
C_6_H_16_BN_4_NaO_5_	3	292–855	341,426, −518,688	C_6_H_2_NO	40.92	40.34	26.39	25.57	1/2Na_2_O.B_2_O_3_	black
Na[B(arg)_2_]	1	112–190	145	2NH_3_	8.48	8.90				white
BAD	2	192–246	225	4NH_3_	18.11	17.98				
C_12_H_24_BN_8_NaO_4_	3	247–801	418,−537, 696,637	2C_6_H_3_NO	55.02	55.58	18.39	17.40	1/2Na_2_O.B_2_O_3_	black

### Effects of BAM and BAD on the Proliferation of B16-F10 Melanoma Cells

**Fig. 4:**
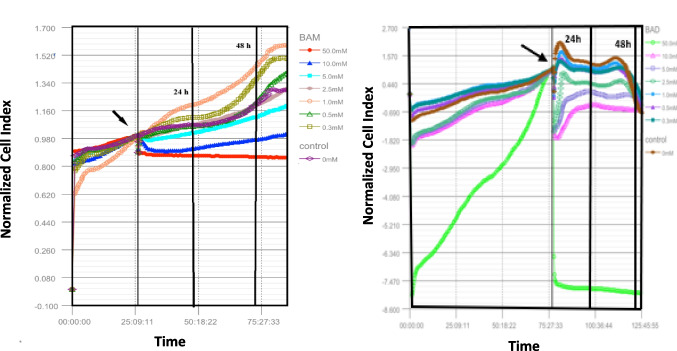
Time curves are normalized cell index. Comparison of B16-F10 cell proliferation after hly application of 50 mM, 10 mM, 5 mM, 2.5 mM, 1 mM, 500 µM and 250 µM BAM and BAD. Arrow indicates the time of substance application

Prior to treatment with BAM and BAD, melanoma cells were cultured in complete growth medium for 24 h. Cells were treated with different concentrations of BAM and BAD (50 mM, 10 mM, 5 mM, 2.5 mM, 1 mM, 500 µM and 250 µM), and viability was monitored for 96 h at 15-min intervals (Fig. 4). Based on the sigmoidal dose–response analysis performed by the iCELLigence™ system, the IC₅₀ values at 24 h were 8.01 mM for BAM and 365 µM for BAD. At 48 h, IC₅₀ values decreased to 5.97 mM for BAM and 51.9 µM for BAD, indicating an increased cytotoxic effect over time. These results indicate that B16-F10 cells are cytotoxically affected by both boron compounds in a dose- and time-dependent manner.

In addition to reporting IC₅₀ values, the real-time cell index curves obtained from the iCELLigence™ system (Fig. 3) show a clear dose- and time-dependent decrease in cell proliferation. For both BAM and BAD, higher concentrations led to an earlier and more pronounced decline in cell index, whereas lower concentrations showed a slower and more gradual reduction. At intermediate concentrations, partial cytotoxicity was observed, with cell index initially increasing before declining over time. These curves visually confirm the time-dependent cytotoxic effects of both compounds on B16-F10 melanoma cells and are consistent with the IC₅₀ values reported at 24 h and 48 h.

### Effects of BAM and BAD Apoptotic and Inflammatory Gene Expression Profiles

**Fig. 5:**
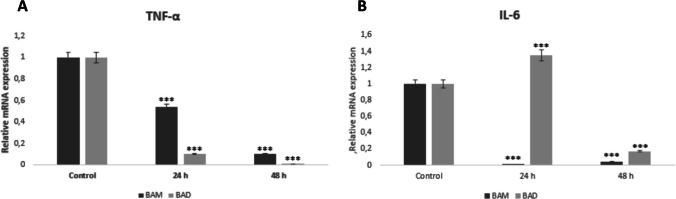
TNF-α gene expression levels following 24 h of 8.01 mM and 48 h of 5.97 mM BAM and 24 h of 365 µM and 48 h of 51.9 µM BAD application (**A**). IL-6 gene expression levels following 24 h of 8.01 mM and 48 h of 5.97 mM BAM and 24 h of 365 µM and 48 h of 51.9 µM BAD application (**B**). BAM: Boron arginine monoester. BAD: Boron arginine diester. *p < 0.05, **p < 0.01, ***p < 0.001

B16-F10 melanoma cells were treated with BAM and BAD at their respective IC₅₀ concentrations, and relative mRNA expression levels of TNF-α, IL-6, Bcl-2, Bax, and p53 were analyzed. TNF-α expression was downregulated to 0.54-, 0.1-, 0.1-, and 0.01-fold of control in cells treated with 24 h of 8.01 mM BAM, 48 h of 5.97 mM BAM, 24 h of 365 µM BAD, and 48 h of 51.9 µM BAD, respectively (Fig. 5A). IL-6 expression decreased to 0.01-, 0.04-, and 0.17-fold of control for 24 h 8.01 mM BAM, 48 h 5.97 mM BAM, and 48 h 51.9 µM BAD, respectively, whereas exposure to 24 h 365 µM BAD resulted in upregulation to 1.35-fold of control (Fig. 5B).

**Fig. 6:**
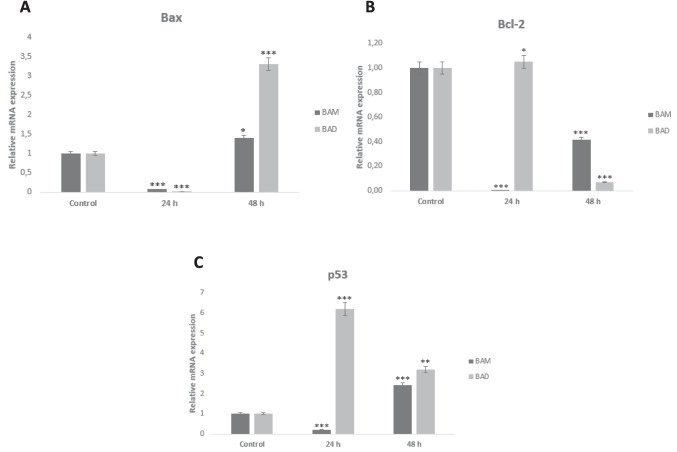
Bax gene expression levels following application of 8.01 mM for 24 h and 5.97 mM BAM for 48 h and 365 µM for 24 h and 51.9 µM BAD for 48 h (**A**). Bcl-2 gene expression levels following application of 8.01 mM for 24 h and 5.97 mM BAM for 24 h and 365 µM for 24 h and 51.9 µM BAD for 48 h (**B**). p53 gene expression levels following application of 8.01 mM for 24 h and 5.97 mM BAM for 24 h and 365 µM for 24 h and 51.9 µM BAD for 48 h (**C**). *p < 0.05, **p < 0.01, ***p < 0.001

Bax expression was downregulated to 0.07- and 0.01-fold of control after treatment with 24 h 8.01 mM BAM and 24 h 365 µM BAD, respectively, while 48 h 5.97 mM BAM and 48 h 51.9 µM BAD upregulated Bax to 1.4- and 3.31-fold of control, respectively (Fig. 6A). Bcl-2 expression was downregulated to 0.00-, 0.42-, and 0.07-fold of control for 24 h 8.01 mM BAM, 48 h 5.97 mM BAM, and 48 h 51.9 µM BAD, respectively, whereas 24 h 365 µM BAD upregulated Bcl-2 to 1.05-fold of control (Fig. 6B). p53 expression decreased to 0.20-fold of control after 24 h 8.01 mM BAM treatment, and increased to 2.44-, 6.2-, and 3.2-fold of control following 48 h 5.97 mM BAM, 24 h 365 µM BAD, and 48 h 51.9 µM BAD treatments, respectively (Fig. 6C).

### Effect of BAM and BAD Application on IL-1β Level in B16-F10 Cells

**Fig. 7:**
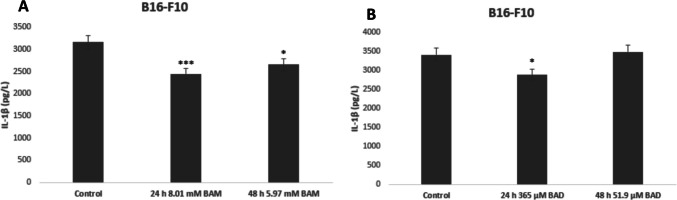
Relative IL-1β levels in the supernatant of B16-F10 cells treated with BAM or BAD at their respective IC₅₀ concentrations for 24 h and 48 h, measured by ELISA and expressed in pg/mL, normalized per well. Data represent mean ± SD (n = 3). Statistical significance was assessed using one-way ANOVA with Tukey's post hoc test (* p < 0.05, *** p < 0.001)

IL-1β levels in the supernatant of B16-F10 cells treated with BAM and BAD were generally lower compared to the control group. Treatment with 8.01 mM BAM for 24 h and 5.97 mM BAM for 48 h significantly reduced IL-1β levels (p = 0.000 and p = 0.045, respectively; Fig. 7A). Treatment with 365 µM BAD for 24 h also resulted in a significant decrease (p = 0.042), whereas 51.9 µM BAD for 48 h did not induce a statistically significant change (Fig. 7B). These results indicate that both BAM and BAD may exert anti-inflammatory effects in B16-F10 cells. Statistical analysis was performed using one-way ANOVA followed by Tukey's post hoc test, with n = 3 biological replicates per group. Asterisks indicate significance (* p < 0.05, *** p < 0.001).

### Effect of BAM and BAD Application on IL-10 Level in B16-F10 Melanoma Cells

**Fig. 8:**
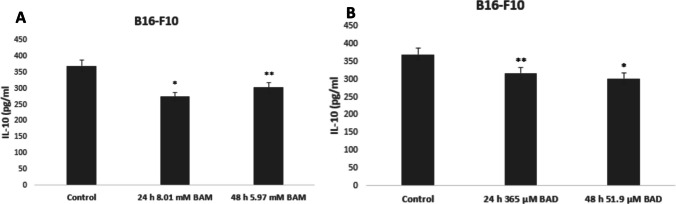
IL-10 levels in B16-F10 melanoma cells. 24 h of 8.01 mM and 48 h of 5.97 mM BAM application (**A**). 24 h of 365 µM and 48 h of 51.9 µM BAD application (**B**). *p < 0.05, **p < 0.01, ***p < 0.001

IL-10 levels in B16-F10 melanoma cells treated with BAM and BAD were generally lower compared to the control group. Treatment with 8.01 mM BAM for 24 h and 5.97 mM BAM for 48 h significantly reduced IL-10 levels (p = 0.007 and p = 0.035, respectively; Fig. 8A). Treatment with 365 µM BAD for 24 h and 51.9 µM BAD for 48 h also significantly decreased IL-10 levels (p = 0.009 and p = 0.014, respectively; Fig. 8B). Statistical analysis was performed using one-way ANOVA followed by Tukey's post hoc test, with n = 3 biological replicates per group. Asterisks indicate significance (* p < 0.05, ** p < 0.01).

### Effect of BAM and BAD Application on Bcl-2 Levels in B16-F10 Cells

**Fig. 9:**
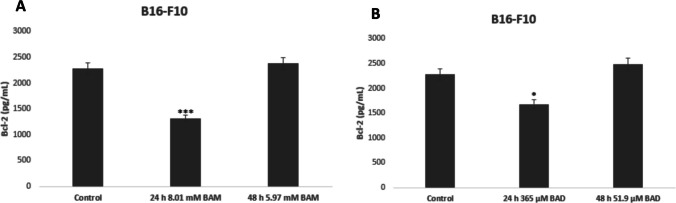
Relative Bcl-2 protein levels in the supernatant of B16-F10 melanoma cells treated with BAM or BAD at their respective IC₅₀ concentrations for 24 h and 48 h, measured by ELISA, expressed in pg/mL, normalized per well. Data represent mean ± SD (n = 3). Statistical significance was assessed using one-way ANOVA with Tukey's post hoc test (* p < 0.05, *** p < 0.001)

In comparison to control cells, Bcl-2 levels in melanoma cells treated with 8.01 mM BAM for 24 h were significantly lower (p = 0.000; Fig. 9A). Treatment with 5.97 mM BAM for 48 h did not result in a statistically significant change. Similarly, treatment with 365 µM BAD for 24 h significantly decreased Bcl-2 levels (p = 0.045; Fig. 9B), whereas 51.9 µM BAD for 48 h had no significant effect. As Bcl-2 is primarily an intracellular anti-apoptotic protein, changes in secreted Bcl-2 levels in the supernatant should be interpreted with caution; while reduced levels may reflect altered cellular responses, they do not directly confirm apoptosis induction.

### Effect of BAM and BAD Application on Annexin V Levels in B16-F10 Melanoma Cells

**Fig. 10:**
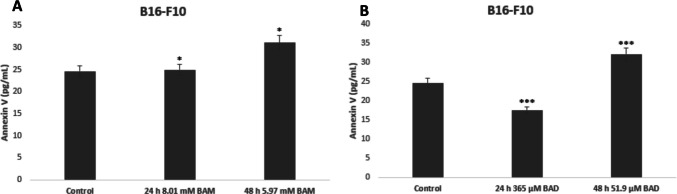
Annexin V levels in B16-F10 melanoma cells after treatment with BAM (**A**) and BAD (**B**) at their respective IC₅₀ concentrations for 24 h and 48 h, measured by ELISA, expressed in pg/mL, using cell lysates. Data represent mean ± SD (n = 3). Statistical significance was assessed using one-way ANOVA with Tukey's post hoc test (* p < 0.05, *** p < 0.001)

Annexin V levels in B16-F10 melanoma cells treated with BAM and BAD were significantly increased compared to untreated control cells in the groups treated with 8.01 mM BAM for 24 h (p = 0.036), 5.97 mM BAM for 48 h (p = 0.017), and 51.9 µM BAD for 48 h (p = 0.000) (Fig. 10). These increases in Annexin V are consistent with cellular responses to cytotoxic treatment; however, as IC₅₀ concentrations were used, this does not directly indicate a specific pro-apoptotic effect. Statistical analysis was performed using one-way ANOVA followed by Tukey's post hoc test, with n = 3 biological replicates per group.

### Antimicrobial Effects of BAM and BAD Application

The MIC and MBC values of BAM and BAD components against selected Gram-positive and Gram-negative bacterial strains are summarized in Table 4. According to the results, the lowest MIC values were observed for Escherichia coli ATCC 25922 and Staphylococcus aureus ATCC 43300 (3.12–12.5 µg/mL), while the lowest MBC values were determined for the same strains (12.5–50 µg/mL). DMSO was used as a solvent, with a final concentration ≤ 1%, and did not affect bacterial growth (solvent control). Positive controls included wells with inoculum only (growth expected), and negative controls included medium only (sterility control). No reference antibiotics were included in this study. MIC and MBC were defined as the lowest concentrations that inhibited visible growth or completely killed bacteria, respectively.

**Table 4:** Antimicrobial results of gram positive and gram negative bacteria

Strain	Minimum Inhibitory Concentration (MIC)		Minimum Bactericidal Concentration (MBC)	
	**BAM**	**BAD**	**BAM**	**BAD**
**Methicillin-resistant *Staphylococcus aureus* (MRSA) (ATCC 43300)**	6.25 µg/ml	12.5 µg/ml	25 µg/ml	50 µg/ml
***Staphylococcus epidermidis*** **ATCC 35984**	3.12 µg/ml	3.12 µg/ml	25 µg/ml	25 µg/ml
***Acinetobacter baumannii*** **ATCC 19606**	6.25 µg/ml	12.5 µg/ml	25 µg/ml	50 µg/ml
***Enterococcus faecalis*** **ATCC 29212**	12.5 µg/ml	3.12 µg/ml	25 µg/ml	12.5 µg/ml
***Escherichia coli*** **ATCC 25922**	6.25 µg/ml	12.5 µg/ml	50 µg/ml	50 µg/ml
***Proteus vulgaris*** **ATCC 6897**	6.25 µg/ml	12.5 µg/ml	25 µg/ml	25 µg/ml
***Klebsiella pneumoniae*** **ATCC 700603**	6.25 µg/ml	6.25 µg/ml	25 µg/ml	25 µg/ml

Minimum inhibitory concentration (MIC) and minimum bactericidal concentration (MBC) of BAM and BAD components against selected Gram-positive and Gram-negative bacteria. Values are expressed in µg/mL, determined by the microdilution method, with n = 3 replicates per strain. MIC is defined as the lowest concentration inhibiting visible bacterial growth; MBC is defined as the lowest concentration at which no growth was observed upon subculturing. DMSO (≤ 1%) was used as solvent control; positive controls contained inoculum only, and negative controls contained medium only.

### Oxidative Stress

**Fig. 11:**
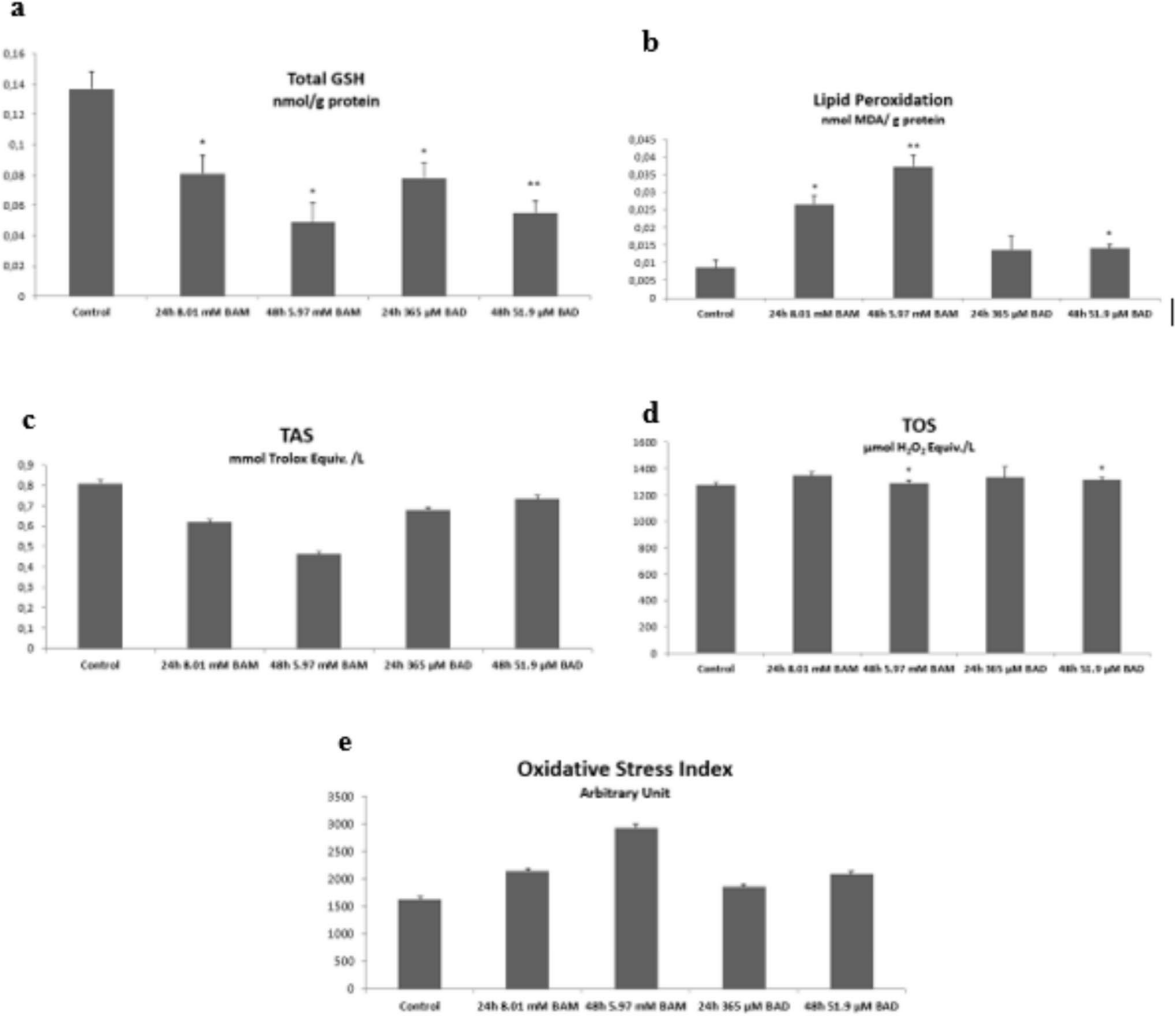
Total Glutathione (GSH) level (**a**), Malondialdehyde (MDA) level (**b**), Total Antioxidant (TAS) level (**c**), Total Oxidant (TOS) level (**d**) and Oxidative Stress Index (OSI) level (**e**) results in melanoma cells treated with 8.01 mM BAM for 24 h and 5.97 mM BAM for 48 h and 365 µM BAD for 24 h and 51.9 µM BAD for 48 h

B16-F10 melanoma cells treated with BAM and BAD at their respective IC₅₀ concentrations exhibited changes in oxidative stress markers, although high cell death (> 50%) may confound these measurements. Reduced GSH levels were statistically significant in most groups (*p < 0.05), while MDA levels were significantly increased in all groups except 24 h BAD treatment (*p-values = 0.032–0.000). TOS was significantly elevated at 48 h for both BAM and BAD (p < 0.01), whereas TAS and OSI changes were not statistically significant (Fig. 11). These results indicate that BAM and BAD induced signs of oxidative stress (reduced GSH, elevated MDA and TOS at 48 h), while TAS and OSI changes were not significant.

Statistical analysis was performed using one-way ANOVA followed by Tukey’s post hoc test, with n = 3 biological replicates per group. Exact p-values are reported in the figure. Data are presented as mean ± SD, with significance indicated as *p < 0.05, **p < 0.01, ***p < 0.001.

Values are expressed per well, normalized to control, and represent mean ± SD (n = 3). Statistical significance was assessed by one-way ANOVA with Tukey’s post hoc test.

*p < 0.05, **p < 0.01, ***p < 0.001.

## Discussion

Boron-containing compounds have attracted increasing attention as potential therapeutic agents in cancer treatment due to their multifaceted biological activities [24, 39–41]. In addition to their anticancer effects, boron derivatives have demonstrated antimicrobial and antibiofilm properties [40, 42]. In the present study, the biological effects of newly synthesized boron arginine monoester (BAM) and boron arginine diester (BAD) were investigated in B16-F10 melanoma cells, focusing on cytotoxicity, apoptosis, inflammation, oxidative stress, and antimicrobial activity.

Consistent with previous studies reporting low cytotoxicity of boron-based compounds in melanoma models [43, 44], Chiu et al. [43] evaluated the cytotoxicity of borophenylalanine (BPA) in B16-F10 melanoma cells and reported no statistically significant toxicity, with cell viability remaining at approximately 90% after 48 h of incubation across BPA concentrations of 15.6–250 μg/mL, BAM and BAD exhibited relatively low toxicity, with IC₅₀ values of 5.97 mM and 51.9 µM at 48 h, respectively. These findings suggest that both compounds may possess a favorable safety profile. However, further studies using extended exposure times and broader concentration ranges are required to define their cytotoxic potential.

Inflammatory processes play a critical role in melanoma progression. Previous studies have shown that boron derivatives can modulate pro-inflammatory cytokines such as TNF-α and *IL-6* [45–47]. Kısacam et al. [48] reported the positive effects of boron on inflammation in their in vivo study with calcium fructoborate, a boron derivative. In agreement with these findings, our results demonstrated that BAM and BAD significantly reduced TNF-α and IL-6 expression levels compared to control cells. This indicates that these compounds may exert anti-inflammatory effects, which could contribute to their therapeutic potential in melanoma.

It has been established that malignant melanoma exhibits widespread expression of the Bax protein [49]. Other studies have shown that high expression of Bcl-2 is not associated with the progression of malignant melanoma [50]. Apoptosis is a key mechanism in cancer therapy, and the Bax/Bcl-2 ratio is widely used as an indicator of apoptotic sensitivity. In this study, BAM and BAD increased Bax expression while decreasing Bcl-2 expression, resulting in an elevated Bax/Bcl-2 ratio. These findings are consistent with previous reports demonstrating apoptosis induction by boron-based therapies in melanoma models [51]. Identification of p53 and p63, along with Bax and Bcl-2 rearrangements of applicability, is therefore an area of interest for understanding their mechanism in inducing apoptosis in cancer cells [52]. Studies on the cytotoxic and apoptosis-inducing effects of boron derivatives on small cell lung cancer [53], glioblastoma [54], HepG2 [54] cells indicate that these compounds may be a potential option in cancer treatment. Furthermore, the observed upregulation of p53 expression suggests that these compounds may activate p53-mediated apoptotic pathways. Together, these results indicate that BAM and BAD promote apoptosis through mitochondrial and p53-associated mechanisms.

The effects of boron derivatives on immune response modulation were further supported by changes in cytokine production in macrophages. Our results demonstrated concentration- and time-dependent alterations in IL-10 and IL-1β levels, indicating that these compounds may influence immune regulation. Differences observed compared to previous studies [55, 56] suggest that these effects may depend on experimental conditions, including dose and exposure duration.

Some Gram-negative (A. baumannii, P. aeruginosa, E. coli and K. pneumoniae) and Gram-positive (MRSA, E. faecalis) bacteria, which are common causes of health-related infections, are the cause of sepsis and cause serious problems by developing resistance to many antibiotics [57, 58]. In addition to their anticancer effects, BAM and BAD exhibited notable antimicrobial activity against both Gram-positive and Gram-negative bacteria. The observed MIC and MBC values were comparable to or lower than those reported for other boron-based compounds [40, 42, 59], supporting their potential as alternative agents against antibiotic-resistant pathogens. The higher activity of BAM compared to BAD in most tested strains further highlights the importance of structural differences in determining antimicrobial efficacy.

Oxidative stress is a critical factor in cancer cell survival and death. Cancer cells often maintain elevated reactive oxygen species (ROS) levels while simultaneously enhancing antioxidant defenses [60, 61]. In a study by Yalcin et al., when increasing doses of boric acid were applied to Leydig cells, GSH levels did not change at low doses but decreased at high doses [62]. In our earlier study, treatment of hepatocellular carcinoma cells with BGM and BGD did not result in a significant change in GSH levels [59]. In contrast, in the present study, treatment of B16-F10 melanoma cells with BAM and BAD at IC₅₀ concentrations (24 and 48 h) led to a significant decrease in GSH levels compared to the control (p < 0.05). This discrepancy may be attributed to several factors, including compound-specific differences in chemical structure, variations in applied dose ranges, and cell-type–dependent responses. Structural differences between BAM/BAD and BGM/BGD may influence intracellular redox balance and antioxidant defense mechanisms. In addition, melanoma and hepatocellular carcinoma cells are known to differ in their metabolic profiles and oxidative stress responses, which may contribute to the observed variation in GSH levels. Furthermore, the use of IC₅₀ concentrations in the present study may have enhanced oxidative stress, leading to GSH depletion. However, the exact mechanisms underlying these differences cannot be fully elucidated based on the current data and require further investigation. MDA is essentially an unstable byproduct of lipid peroxidation, a highly cytotoxic indicator of lipid peroxidation [63]. It also serves as a co-carcinogen and tumor promoter. The device has established a significant relationship between increased serum MDA levels and decreased antioxidant parameters, and has been proven in many types of cancer, including colorectal, oral, prostate, gastric, lung and breast [64]. Increased MDA levels were observed in prostate cancer cells treated with boric acid [65] and in hepatocellular carcinoma cells treated with boron compounds [59]. MDA levels increased in all treatment groups in this investigation, despite the 24-h BAD group showing no statistically significant rise.

It is stated that healthy amounts of boron reduce the risk of cancer and DNA damage and increase antioxidant levels [66]. It is important to note that the effects of boron compounds on oxidative balance appear to be cell type-dependent, as inconsistent findings have been reported across different cancer models [67, 68]. Therefore, further studies are needed to clarify the underlying mechanisms and to determine optimal therapeutic conditions.

Overall, the present study demonstrates that BAM and BAD exhibit low cytotoxicity, while exerting significant apoptotic, anti-inflammatory, antimicrobial, and pro-oxidant effects in melanoma cells. These multifunctional properties highlight their potential as promising candidates for melanoma therapy. However, further in vitro and in vivo studies are required to validate these findings and to elucidate their long-term therapeutic efficacy and safety.

## Limitations of the Study

This study evaluated the biological effects of newly synthesized BAM and BAD compounds in the B16-F10 melanoma cell line. The obtained IC₅₀ values indicate that these compounds possess measurable biological activity. Notably, the lower IC₅₀ value observed for BAD compared to BAM suggests that the degree of esterification may influence cellular responses. However, further studies are required to elucidate these differences through detailed mechanistic and statistical analyses.

A major limitation of this study is the absence of selectivity assessments in non-cancerous cell lines, which limits the evaluation of potential toxicity in normal tissues. Additionally, the in vitro nature of these experiments prevents conclusions regarding systemic safety. Potential off-target effects, pharmacokinetics, and biodistribution were not evaluated. Therefore, these results should be interpreted as preliminary in vitro findings, providing a foundation for future studies, including in vivo investigations, to fully assess the therapeutic potential and safety profile of BAM and BAD.

## Conclusion

In conclusion, BAM and BAD demonstrated promising therapeutic potential in B16-F10 melanoma cells by inhibiting cell proliferation in a dose-dependent manner and modulating key biological processes, including apoptosis and inflammation. To the best of our knowledge, this study represents one of the first investigations of boron ester compounds in a melanoma model, highlighting their potential as novel anticancer agents.

The observed biological activities suggest that these compounds may be effective against aggressive cancers such as melanoma. However, further studies involving different cell lines, detailed molecular analyses, and in vivo models are required to fully elucidate their mechanisms of action and evaluate their long-term therapeutic efficacy and safety.

## Data Availability

No datasets were generated or analysed during the current study.
